# Supplementary material for: Effective population size in field pea
Source: BMC Genomics. 2024 Jul 16;25:695. doi: 10.1186/s12864-024-10587-6 (PMC11251210; doi:10.1186/s12864-024-10587-6)
Supplement: Supplementary file 1 — Supplementary Material 1. [file 12864_2024_10587_MOESM1_ESM.pdf]

Supplementary Figures

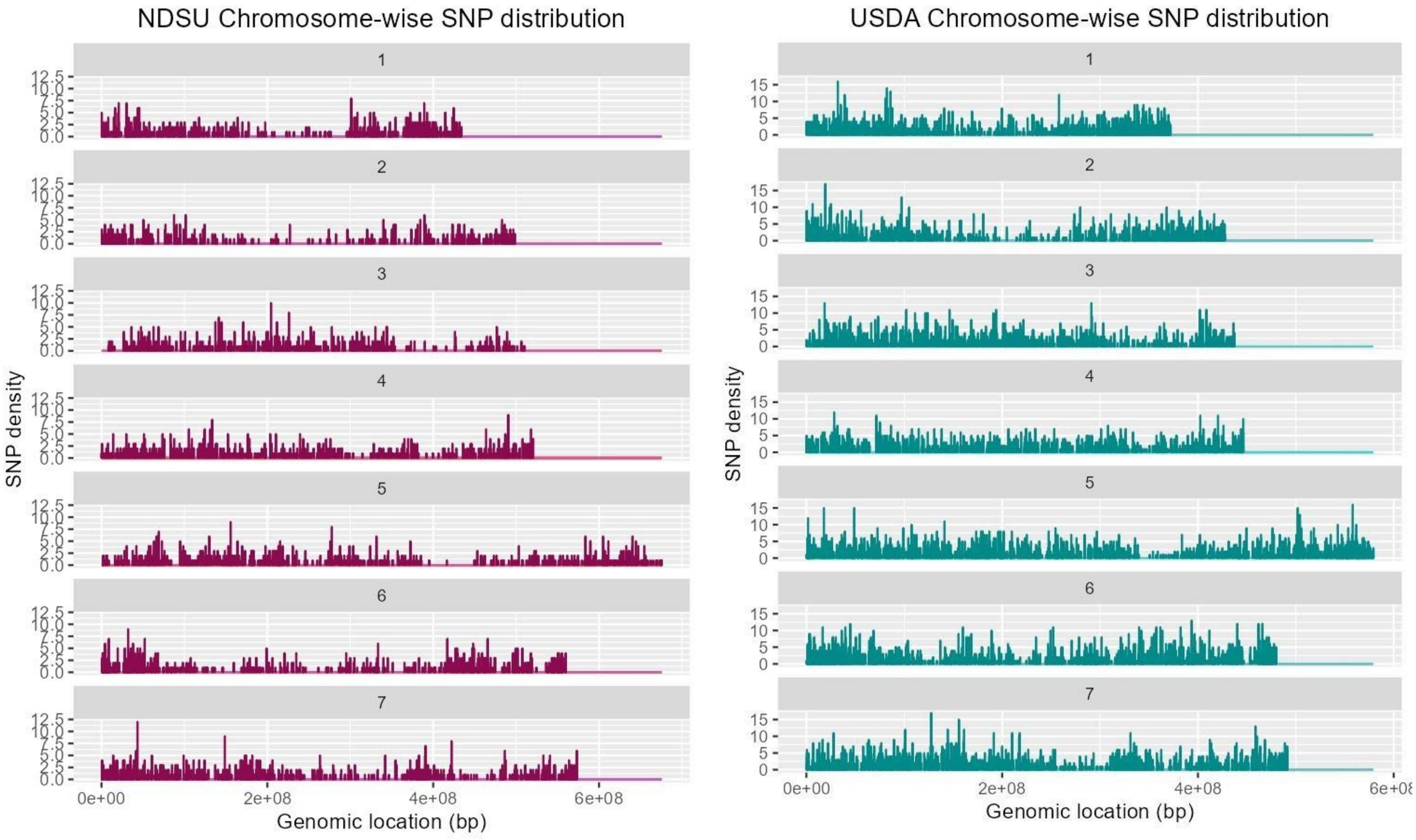

Supplementary Figure 1: SNP density of NDSU set and USDA set, x-axis is the genomic location (bp) and y-axis is the density. The empty lines in the small chromosomes represents that there are no markers available in those regions.

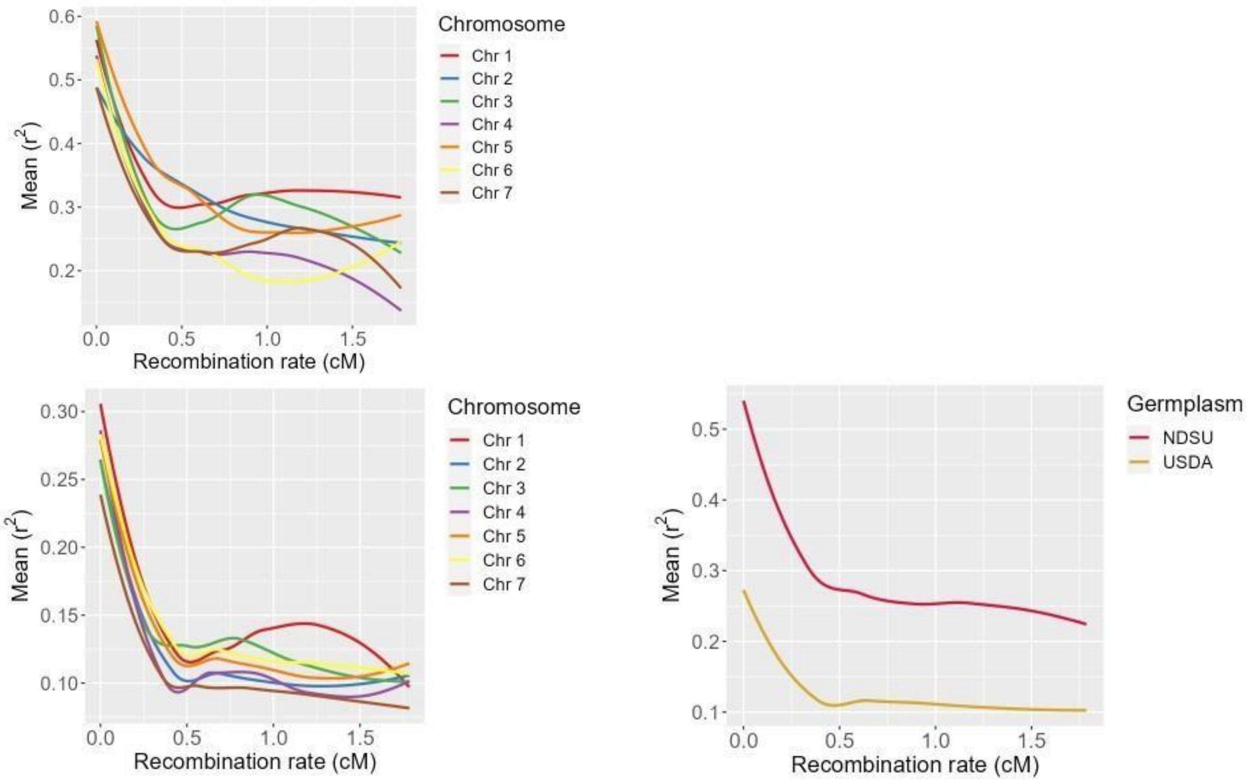

Supplementary Figure 2: Genome-wide (C) and Chromosome-wide linkage disequilibrium decay in the NDSU set (A) and USDA set (B) with mean of r^2 (y-axis) and recombination rate (cM) (x-axis)
